# Changes in the site distribution of malignant melanoma in South East Scotland (1979–2002)

**DOI:** 10.1038/sj.bjc.6603612

**Published:** 2007-02-13

**Authors:** M Mowbray, D L Stockton, V R Doherty

**Affiliations:** 1 Department of Dermatology, University of Edinburgh, The Royal Infirmary of Edinburgh, Level 1 The Lauriston Building, Lauriston Place, Edinburgh EH3 9HA, UK; 2 Public Health Information Programme Manager, NHS National services Scotland, 1st Floor Gyle Square, 1 South Gyle Crescent, Edinburgh EH12 9EB, UK

**Keywords:** aetiology, body site, epidemiology, malignant melanoma, Scotland, sunlight exposure

## Abstract

Scottish Melanoma Group (SMG) data on 2790 melanoma (MM) cases in South East Scotland over a 24-year time period were analysed in four periods each of 6 years duration grouped into frequently exposed, intermittently exposed, and always covered sites. Incidence increased significantly over time with females having a higher incidence rate than males. In both sexes, the proportion of cases seen on the posterior trunk and arm increased significantly (*P*<0.001), but declines were seen in the proportion of leg tumours in males (*P*=0.09) and of head tumours in females (*P*=0.011). Although the proportion of cases decreased for certain sites, the actual MM incidence increased at all sites. A significant increase in incidence occurred at usually and always covered sites (*P*<0.001 and *P*<0.001, respectively) in females and at usually covered sites in males (*P*<0.001).

The increase in incidence of primary cutaneous malignant melanoma (MM) in Caucasian populations worldwide over the past two decades is well documented. Differences are emerging in the patterns of change in incidence rates seen between countries. In ‘high incidence regions’ such as Australia (Green *et al*, 1993; Marrett *et al*, 2001), the annual age-standardised incidence rates for MM have always been higher in men than in women and the rate of increase in incidence has been steeper in men over the past 25 years. In more moderate risk areas such as Canada (Bulliard *et al*, 1999) and the USA (Chen *et al*, 1994), the rate of increase in the incidence of invasive melanomas in males has been so much greater than that seen in females that male incidence rates have overtaken those in women. Scotland is a relatively ‘low incidence region’, with actual incidence rates of 8–10 per 100 000 population in 1998 compared with 30–45 per 100 000 population in New South Wales in 1995 (Marrett *et al*, 2001). Incidence rates in Scotland for MM have always been, and remain, higher in women. Over the last 25 years, however, the rate of increase has been much greater for men in Scotland too.

Melanoma incidence in Australasia started to level off in those born after 1945–1950 (Bulliard and Cox, 2000; Marrett *et al*, 2001) with a plateau since the late 1980s, and a similar effect has also been reported from Canada for those born after 1944 (Bulliard *et al*, 1999). No birth cohort effect is yet seen in Scottish data where incidence continues to rise in men, although there is some levelling of incidence rates in younger women since 1994 (Mackie *et al*, 2002).

We have examined the site distribution of invasive MM in South East (SE) Scotland over a 24-year period (1979–2002) with the hope of improving our knowledge of the effect of sun exposure patterns on these cancers.

## MATERIALS AND METHODS

Data from the multi-disciplinary Scottish Melanoma Group (SMG), set up in 1978 to gather detailed clinical, pathological, treatment and follow-up information in all cases of primary invasive cutaneous melanoma in Scotland, were used to identify all National Health Service and private sector patients with invasive MM (Clark level 2 or deeper) in the years 1979–2002. Registrations are crosschecked with pathology departments and annually with the Scottish Cancer Registry with follow-up information at 5, 10, 15 and 20 years after diagnosis. The SMG covering a relatively stable population of five million is one of very few population-based melanoma databases in the world.

We have analysed the details of all 2790 patients presenting with invasive MM in SE Scotland SMG database (population 1.2 million) over a 24-year time period from 1979 to 2002, in four equal periods each of 6 years. For historical reasons, data from the Highlands and Western Isles were included, although for simplicity, we still refer to the study region as SE Scotland.

The sites of the MM were grouped into frequently exposed (face, scalp, neck, ears, dorsal foot, lip, lower arm, dorsal hand and lower leg females), usually covered/intermittently exposed (trunk above waist, upper arm, upper leg, lower leg males) and always covered (trunk below waist, subungual hand, subungual toe, palm, sole and mucosal) sites. Unknown sites, tumours on the arm (area unspecified) and the leg (area unspecified) for males were excluded from the exposure group analyses (52/1.9% of cases). Female lower leg was classified as ‘frequently exposed’ and male lower leg as ‘usually covered’; all female data were complete and specified upper/lower leg and hence no females required exclusion from the analysis.

The following characteristics were recorded for all patients:

sex, numbers and proportions of male and female,
age (<50, 50–69, ⩾70),
site (as above),
tumour thickness (Breslow >0–0.74, 0.75–1.49, 1.5–3.49, ⩾3.5 mm, Clarks level 2–5 and unknown).

These features were compared across the four periods.

Differences in proportions were tested using the *χ*^2^ statistic. Changes in incidence rates over time were assessed using Poisson regression. Population estimates for SE Scotland (including Highland, Lothian, Fife, Borders and Western Isles Health Boards) from the General Registrars Office for Scotland (GROS) were used to calculate age-standardised incidence rates (standardised to the European population).

## RESULTS

### Overall incidence rates

**Figure 1:**
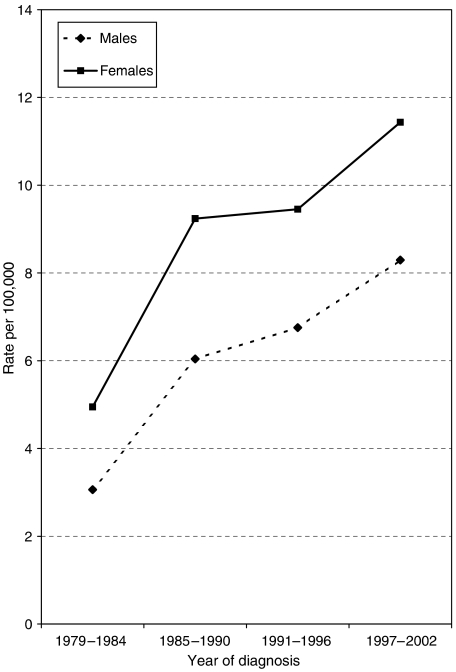
Age-standardised incidence rates of melanoma in SE Scotland, 1979–2002.

Over the 24 years (1979–2002), 1759 MM patients (63%) were female and 1031 (37%) male. The number of cases has increased significantly over time from 357 cases in the first period to 976 in the final period, 1997–2002 (Figure 1). The higher incidence in females persisted throughout the study period, rates increasing in both sexes similarly (Figure 1).

### Sites of occurrence

**Figure 2:**
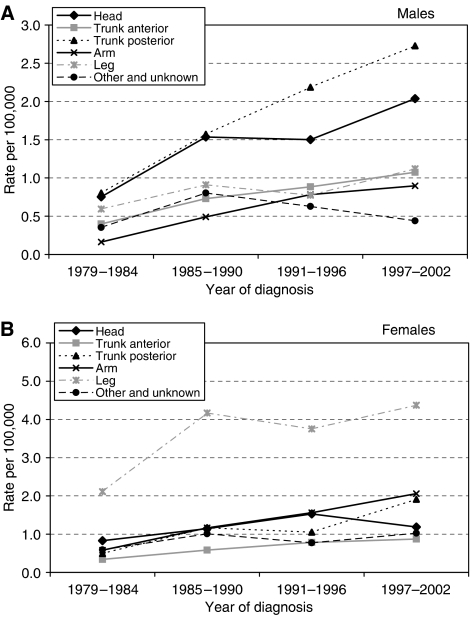
Age standardised (European) incidence of melanoma in SE Scotland at different sites, 1979–2002. (**A**) Males and (**B**) Females.

Overall, the most common sites were the posterior trunk and head for males and the leg and arm for females (Figures 2A and B); less common sites were grouped together as ‘other and unknown’ and of the total 2790 lesions, only 16 (0.57%) were classified as unknown. For both males and females, the proportion of cases seen on the posterior trunk and arm increased significantly (*P*<0.001) over the study period; no change in proportion was seen for the anterior trunk. In males, a nonsignificant decline in the proportion of leg tumours was seen (*P*=0.09), and of head tumours in females (*P*=0.011). The proportion of other and unknown sites decreased in both sexes over time (Table 1a and b).

Although the proportions declined at certain sites, the actual incidence (number) of melanoma increased at all sites over the time period, and significantly so with the exception of other and unknown sites for males and head sites for females (Figures 2A and B).

### Exposure patterns

**Figure 3:**
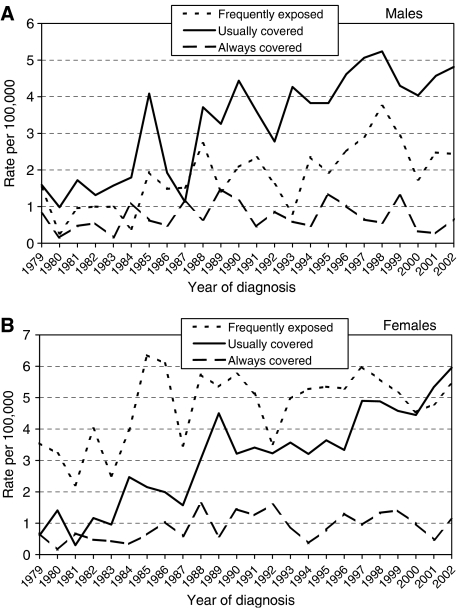
Age standardised (European) incidence of melanoma in SE Scotland at frequently exposed, usually covered and always covered sites, 1979–2002. (**A**) Males and (**B**) Females.

One thousand two hundred and ninety-six cases were at frequently exposed sites, 1125 at usually covered sites and 317 at always covered sites. A significant increase in incidence over time was seen in females at usually and always covered sites (*P*<0.001 and *P*<0.001, respectively). A similar increase at usually covered sites was seen in males (*P*<0.001), with no change at always covered sites for males (Figures 3A and B).

### Age/birth cohort effect

There was no evidence of a significant deviation in the pattern of incidence over time by age group; the trend across all sites for all age groups was of an increase in incidence rates. The only exception was anterior chest lesions in women where incidence decreased over time for those greater than 70 years old but increased for younger age groups (data not shown).

### Tumour thickness and histology

A significant decrease in Breslow thickness was seen over the 24-year time period (Table 2), for each site except the head and neck. In the first time period, the proportion of thin melanomas was 41% (Breslow <1.49 mm) with an increase to 67% by the fourth time period. In the first time period, the distribution of histological types was superficial spreading (49%), nodular (25%) and lentigo maligna (15%). The same three types were also most frequent in the fourth time period with distribution markedly changed to superficial spreading (70%), nodular (10%) and lentigo maligna (12%).

## DISCUSSION

A number of different studies worldwide have analysed site-specific changes of MM in large populations over extended time periods. In contrast to trends in overall incidence rates, the patterns of change in the site distribution of MM seem in general to be similar between higher and lower incidence regions. Overall, the commonest sites in males are the trunk (Gallagher *et al*, 1990; Thorn *et al*, 1990; Chen *et al*, 1994; Bulliard *et al*, 1999; Bulliard, 2000; Bulliard and Cox, 2000; Marrett *et al*, 2001; Swerdlow, 2001; MacKie *et al*, 2002; Stang *et al*, 2006) followed by the head and neck (Chen *et al*, 1994; Bulliard, 2000; MacKie *et al*, 2002). In females, the commonest site for invasive MM is the lower limb (Gallagher *et al*, 1990; Thorn *et al*, 1990; Chen *et al*, 1994; Bulliard, 2000; Bulliard and Cox, 2000; Marrett *et al*, 2001; MacKie *et al*, 2002; Stang *et al*, 2006). Some studies also suggest a fall in the incidence of lower limb lesions in males (Chen *et al*, 1994; Bulliard *et al*, 1999) and head and neck lesions in females (Gallagher *et al*, 1990; Thorn *et al*, 1990; Green *et al*, 1993; Chen *et al*, 1994; Bulliard *et al*, 1999; Bulliard and Cox, 2000).

SE Scotland follows the world trends with an increase in the incidence of invasive MM in both sexes during the past 24 years. A number of reasons for this increase have been postulated, such as changes in diagnostic histopathologic criteria, improved case ascertainment and increased public awareness with a subsequent increase in presentation. Although such factors no doubt account for some of the increase observed, evidence would suggest that they do not make a great contribution (van der Esch *et al*, 1991; Swerdlow, 2001). Some countries are reporting stabilisation of incidence rate rises either generally or in younger female patients. It has been suggested that this may be due to primary prevention campaigns (Chen *et al*, 1994; Gaudette and Gao, 1998; Bulliard *et al*, 1999; Bulliard and Cox, 2000; Marrett *et al*, 2001), although some would argue that it is too early to be seeing falls in melanoma incidence as a result of such education (Bulliard and Cox, 2000). Likewise, any fall could reflect increased sunscreen use, which has become commonplace in the past 25 years. In the late 1970s to early 1980s, the UVB protection offered by most commercially available sunscreens was low (SPF 4–10), with little in the way of available UVA filters. Only in the past 10 years have high-protection sunscreens (SPF 15–60) become more widely available, but the levelling off in MM incidence in Scotland has not been seen elsewhere.

We have reported on changes in site from a lower incidence temperate area, where intermittent bursts of sun exposure sometimes with high intensity is the norm, this behaviour having increased over the past 15 years (Swerdlow, 2001). The site trends that we observed are similar to those seen worldwide, with a proportional increase in lesions on the posterior trunk and arms for both sexes, a proportional decrease in leg lesions in males and of head and neck lesions in females. In addition to an increase in intermittent exposure, clothing changes such as the wearing of briefer clothing during recreational exposure may also have an effect.

The potential contribution made by different exposure patterns is further highlighted by our data on changes at ‘frequently exposed’ compared to ‘usually covered’ sites. A significant increase was seen in the proportion of MM at ‘usually covered’ sites in women, with a similar but nonsignificant change in men. This increase in MM incidence over time may in part be due to an increase in intermittent sun exposure, as reported from a comprehensive meta-analysis of all MM studies (Gandini *et al*, 2005).

In our analysis, we did not adjust for body surface area. It is reassuring, therefore, that the findings in comparable studies that did so are similar to ours (Green *et al*, 1993; Bulliard, 2000; Stang *et al*, 2006).

The SMG data set is well known for being more comprehensive than data available from general cancer registration. It has certainly allowed us to build a detailed picture of the changes in site distribution of invasive malignant melanoma in SE Scotland over time. As it was retrospective, it was not possible to assess skin type or past sun exposure details for the patients. On a day-to-day basis, Scotland is certainly a ‘low-exposure’ region for occupational and recreational purposes. However, intermittent exposure in the form of regular holidays in the sun has become increasingly common over the last 20–30 years. The change in site distribution that we have noted, with more melanomas arising on intermittently exposed skin, may be a reflection of changing lifestyles and fashion in the last few decades.

## Figures and Tables

**Table 1:** Distribution of tumour site by year and sex (*N* and %), (a) males and (b) females

	**1979–1984**	**1985–1990**	**1991–1996**	**1997–2002**	**Total**
*(a) Males*					
Head	31	62	66	96	255
	25.83	25.31	22.92	25.40	24.73
Anterior trunk	16	31	37	49	133
	13.33	12.65	12.85	12.96	12.90
Posterior trunk	29	64	92	123	308
	24.17	26.12	31.94	32.54	29.87
Arm	7	20	34	40	101
	5.83	8.16	11.81	10.58	9.80
Leg	23	36	33	49	141
	19.17	14.69	11.46	12.96	13.68
Other and unknown	14	32	26	21	93
	11.67	13.06	9.03	5.56	9.02
Total (*N*)	120	245	288	378	1031
%	100.00	100.00	100.00	100.00	100.00
					
*(b) Females*					
Head	49	70	95	79	293
	21	16	20	13	17
Anterior trunk	17	26	37	41	121
	7	6	8	7	7
Posterior trunk	21	51	50	93	215
	9	12	10	16	12
Arm	26	54	74	102	256
	11	12	15	17	15
Leg	91	184	181	221	677
	38	42	37	37	38
Other and unknown	33	53	49	62	197
	14	12	10	10	11
Total (*N*)	237	438	486	598	1759
%	100	100	100	100	100

**Table 2:** Breslow group by year (*P*<0.001)

**Breslow (mm)**	**1979–1984**	**1985–1990**	**1991–1996**	**1997–2002**	**Total**
>0–0.74	96	264	298	427	1085
	27	39	39	44	39
0.75–1.49	49	126	143	222	540
	14	18	18	23	19
1.5–3.49	96	155	177	171	599
	27	23	23	18	21
3.5+	112	119	137	151	519
	31	17	18	15	19
Unknown	4	19	19	5	47
	1	3	2	1	2
Total (*N*)	357	683	774	976	2790
(%)	100	100	100	100	100
